# Serpin–4 Facilitates Baculovirus Infection by Inhibiting Melanization in Asian Corn Borer, *Ostrinia furnacalis* (Guenée)

**DOI:** 10.3389/fimmu.2022.905357

**Published:** 2022-06-09

**Authors:** Jiayue Ji, Dongxu Shen, Shasha Zhang, Lei Wang, Chunju An

**Affiliations:** ^1^ Department of Entomology, College of Plant Protection, China Agricultural University, Beijing, China; ^2^ Jiangsu Key Laboratory of Sericultural Biology and Biotechnology, School of Biotechnology, Jiangsu University of Science and Technology, Zhenjiang, China; ^3^ Key Laboratory of Silkworm and Mulberry Genetic Improvement, Ministry of Agriculture and Rural Affairs, Sericultural Research Institute, Chinese Academy of Agricultural Sciences, Zhenjiang, China

**Keywords:** serpin-4, baculovirus, melanization, inhibition, *Ostrinia furnacalis*

## Abstract

Phenoloxidase (PO)–catalyzed melanization is a vital immune response in insects for defense against pathogen infection. This process is mediated by clip domain serine proteases and regulated by members of the serpin superfamily. We here revealed that the infection of *Autographa californica* multicapsid nucleopolyhedrovirus (AcMNPV) significantly inhibited the PO activity in *Ostrinia furnacalis* hemolymph and induced the expression of *O. furnacalis serpin–4*. Addition of recombinant serpin-4 protein to *O. furnacalis* hemolymph resulted in a great increase of AcMNPV copies. Serpin-4 significantly suppressed the PO activity and the amidase activity in cleaving colorimetric substrate IEAR*p*NA (IEARase activity) of hemolymph. Further experiments indicated it formed covalent complexes with three serine proteases (SP1, SP13 and SP105) and prevented them from cleaving their cognate downstream proteases *in vitro*. Altogether, *O. furnacalis* melanization restricted AcMNPV replication and serpin-4 facilitated AcMNPV infection by inhibiting serine proteases, SP1, SP13, and SP105 which were all involved in the melanization response.

## Introduction

Insects inevitably encounter various pathogens including viruses during their lifetime, but they still survive in a microbe–rich natural environment (1). This is mainly attributed to the powerful innate immune system in insects, especially in the case most insects lack a typical adaptive immune system (2, 3). Among insect innate immune responses, melanization is a prominent humoral reaction and leads to the synthesis of melanin (4). A number of studies have shown that melanization, combined with other immune mechanisms such as antimicrobial peptide (AMP) production, provides defense against bacteria (5), fungi (6, 7), and parasites (8). Some research revealed that melanization was also effective in resistance against viruses. For example, the melanized tracheal epidermis limited the spread of *Autographa californica* multicapsid nucleopolyhedrovirus (AcMNPV) to hemocytes and other tissues in resistant *Helicoverpa zea* larvae (9). The melanized plasma in *Heliothis virescens* accounted for the inactivation of *H. zea* single capsid nucleopolyhedrovirus (HzSNPV) *in vitro* (10). Hemolymph melanization in Lepidoptera was correlated with antiviral activity against *Microplitis demolitor* bracovirus (MdBV), *Lymantria dispar* MNPV and *Helicoverpa armigera* nucleopolyhedrovirus (HearNPV) (11, 12).

The melanization reaction is mediated by multiple activating and regulating factors. During insect melanization, a series of serine proteases (SPs) are sequentially activated upon infection and culminate in the activation of prophenoloxidase activating proteinase (PAP). Activated PAP further converts inactive prophenoloxidase (PPO) to phenoloxidase (PO) (13). The resulting active phenoloxidase catalyzes the oxidation of phenols to quinones which spontaneously polymerize to form melanin (14, 15). This process is strictly regulated by members of the serine protease inhibitor (serpin) superfamily through targeting at specific serine protease(s) (16). Serpins adopt a canonical fold of three β–sheets and up to nine α–helices with a reactive center loop (RCL) exposed at the surface (17, 18). When a serpin interacts with its target serine protease, it is cleaved at the scissile bond in the RCL by the target serine protease and subsequently forms a covalent complex with the target serine protease, which is therefore irreversibly inhibited (18, 19). Many serpins have been reported to regulate insect melanization, such as SRPN1 and SRPN2 in *Aedes agypti* (20), SRPN2 in *Anopheles gambiae* (21), serpin–5, –6, –15 and –32 in *Bombyx mori* (22–25), serpin–5 and serpin–9 in *Helicoverpa armigera* (26), serpin–1, serpin–3, serpin–4, serpin–5, serpin–6, serpin–7, serpin–9, serpin–12 and serpin–13 in *Manduca sexta* (27–30) and SPN40, SPN55 and SPN48 in *Tenebrio molitor* (31).

The inhibition of melanization by serpins has been reported to affect the antibacterial and antiparasitic responses (23, 32, 33). Recent studies revealed that serpins also participated in the antiviral processes in insects. For example, in *H. armigera*, suppression of melanization by serpin–5 and serpin–9 promoted the baculovirus infection (26). In *B. mori*, depletion of *serpin-2* resulted in the decrease of the number of BmNPV genomic DNA copies (34). Expression of a viral serpin *Hesp018* increased the virulence of baculovirus in infected *Trichoplusia ni* larvae, possibly due to its ability to inhibit the activity of host protease involved in PPO activation (35). Comparing with the understanding of the role of serpin in antibacterial response, knowledge about its function in insect antiviral reaction is relatively lacking.

The Asian corn borer, *Ostrinia furnacalis* (Guenée), is an important agricultural pest in many regions of Asia and causes great economic losses (36). The strategy suppressing *O. furnacalis* larvae by the natural enemy such as entomopathogenic virus or fungi has been proposed. Our previous work illuminated partly the molecular and biochemical mechanisms involved in interaction between *O. furnacalis* and entomopathogenic fungi *Beauveria bassiana* (37). Four serine proteases, SP1, SP7, SP13 and SP105 mediated the melanization in *O. furnacalis* upon the infection of *B. bassiana* (38–40). However, knowledge about the crosstalk between *O. furnacalis* and entomopathogenic virus is very incomplete. In this study, we investigated the relationship between AcMNPV infection and *O. furnacalis* melanization, and discovered that *O. furnacalis* serpin-4 facilitated AcMNPV infection by inhibiting its target serine proteases, SP1, SP13, and SP105 which were all involved in the melanization response.

## Materials and Methods

### Insect Rearing and AcMNPV Preparation

Asian corn borers, *O. furnacalis* (Guenée) larvae were reared on an artificial diet at 28°C under a relative humidity of 70–90% and a photoperiod of 16 h of light and 8 h dark. AcMNPV was purchased from Henan Jiyuan Baiyun Industry Co., Ltd. and dissolved in phosphate–buffered saline (PBS).

### Examination of gDNA Copies of AcMNPV in Infected *O. furnacalis* Larvae

To explore the replication process of AcMNPV in its host *O. furnacalis* larvae, each fifth–instar day 0 larvae were injected with 1 μL of AcMNPV (2.5 × 10^4^ polyhedral inclusion body (PIB)/μL) or PBS as a control (2 larvae/treatment). Each treatment was performed 3 times individually. After 1, 6, 12 and 18 h, the total genomic DNA (gDNA) was individually extracted with the Genomic DNA Extraction Kit Ver.5.0 (TaKaRa, Japan) following the manufacturer’s instructions. Specific primers (**Table S1**) were designed to amplify AcMNPV *ODV–e56* which encoded an occlusion–derived virus–specific envelope protein (41). *O. furnacalis rpL8* was used to normalize the expression of *ODV–e56*. qRT–PCR was performed on an Applied Biosystems 7500 Real Time System (Life Technologies™) using SuperReal PreMix Plus (SYBRGreen) (TIANGEN, Beijing, China) with gDNA as a template, according to the manufacturer’s instructions. The thermal cycling conditions for qRT–PCR were 95°C for 15 min followed by 40 cycles of 95°C for 10 s, 60°C for 30 s and 72°C for 32 s to generate a melting curve. Each qRT–PCR experiment was performed in 3 biological replicates. The relative viral gDNA expression was calculated using the 2^–ΔΔCt^ method.

### Analysis of PO Activity and Expression of Serpin in AcMNPV–Infected *O. furncalis* Larvae

To check whether the replication of AcMNPV in *O. furnacalis* was affected by the melanization response of *O. furnacalis*, we injected 1 μL of AcMNPV at different concentrations (2.5 × 10^3^, 2.5 × 10^4^ and 6 × 10^4^ PIB/μL) into the hemocoel of *O. furnacalis* fifth instar day 0 larvae. Injection of 1 μL of sterile PBS was used as a control. At 1, 3, 6, 9, 12 and 18 h post infection (hpi), 1 µL of hemolymph was collected from individual larva and incubated with 9 µL of saline buffer (20 mM Tris, 150 mM NaCl, pH 8.0) at room temperature for 10 min. Then, PO activity of the reaction mixture was measured using dopamine as the substrate. One unit of PO activity was defined as the amount of enzyme producing an increase in absorbance (Δ*A*_490_) of 0.001 per min.

To check whether the replication of AcMNPV in *O. furnacalis* was related to the expression of serpins which are known inhibitors of insect melanization response, we injected 1 µL of AcMNPV suspension (2.5 × 10^4^ PIB/μL, sterile PBS as a control) into fifth instar day 0 larvae. Three hours later, total RNA was isolated from the whole body of each larva (3 larvae/each group) with TRIzol reagent (TIANGEN, Beijing, China) following the manufacturer’s instructions. One microgram of total RNA from each larva was converted into first–strand cDNA using a FastQuant RT Kit (TIANGEN, Beijing, China) following the manufacturer’s protocol. The cDNA products were diluted 10–fold for use as templates in qRT-PCR. Specific primers were designed based on the cDNA sequences from assembled *O. furnacalis* transcriptome (37) and listed in **Table S1**. qRT–PCR was performed as described above.

### Cloning and Expression Profile Analysis of *O. furnacalis Serpin–4*

Based on the data from “Material and methods 2.3”, *O. furnacalis serpin-4* (37) was selected for further cloning and characterization. Specific primers (**Table S1**) were designed for the amplification of full–length cDNA encompassing the entire reading frame with cDNA from *O. furnacalis* larvae as a template. The products were cloned into the pMD19–T vector, and the nucleotide sequences were confirmed by DNA sequencing. To investigate the transcriptional changes of *serpin–4* during the various developmental stages, total RNA was individually prepared from three different stages including egg, larva, and pupa. To determine the expression patterns of *serpin–4* in different tissues, total RNA samples were isolated separately from the heads, guts, fat bodies, and hemocytes of day 1 fifth–instar larvae. The synthesis of first–strand cDNA and qRT–PCR analyses were performed as described above.

### Production of Recombinant Serpin–4 and GFP Proteins and Preparation of Antiserum Against Serpin–4

To produce recombinant serpin–4 (rserpin-4), a cDNA fragment encoding mature serpin–4 was amplified by PCR using the specific primers listed in **Table S1**. The forward primer included an *Nco* I site, which provided the start codon, followed by one codon for a glycine residue and six codons for histidine residues. The reverse primer contained a stop codon and a *Not* I site. The PCR products were ligated into the pMD19–T vector and then digested with *Nco* I and *Not* I (TaKaRa, Japan). The digested product was subcloned into the same restriction sites of the expression vector pET28a (Novagen). After sequence confirmation, the resulting serpin–4/pET28a plasmids and gifted GFP/pET28a plasmids were used to transform *E. coli* BL21 (DE3) cells, respectively. For recombinant protein expression, a single clone was incubated at 37°C in LB medium containing 50 μg/mL kanamycin. When the OD_600_ of the culture reached 0.8, isopropyl β–D–thiogalactoside was added to a final concentration of 0.1 mM, and recombinant protein was expressed for 13 h at 22°C and 220 rpm. The bacteria were harvested by centrifugation at 3,000 × g for 30 min and resuspended in lysis buffer (50 mM sodium phosphate, 300 mM NaCl and 5 mM imidazole, pH 8.0). The bacteria cells were lysed by sonication, and a cleared clear lysate was obtained by centrifugation. The soluble rserpin–4 or rGFP in the supernatant was purified as described previously (29). Two milligram of the purified serpin–4 was used as an antigen to produce a rabbit polyclonal antiserum (Beijing CoWin Bioscience Co., Ltd). The remaining recombinant protein was stored at –80°C for further use.

The other recombinant proteins, including *O. furnacalis* PPO2, mutated proSP1 (proSP1_Xa_), wild type proSP13, mutated proSP13 (proSP13_Xa_), and mutated proSP105 (proSP105_Xa_) were successfully obtained in our previous work (38, 39, 42). In proSP1_Xa_ and proSP13_Xa_, the cleavage activation site was changed to IEGR to permit its activation by bovine Factor Xa which was commercially available (43).

### Effect of Serpin–4 on AcMNPV Replication and Hemolymph Melanization

To investigate the effect of serpin–4 on AcMNPV replication in *O. furnacalis* plasma (cell–free hemolymph), AcMNPV (1.25×10^4^ PIB) was mixed with 5 μL of plasma from day 0 fifth instar larvae plus 5 μL of rSerpin–4 (1.2 μg/μL) or 5 μL of rGFP (1.2 μg/μL), or 5 μL of 20 mM phenylthiourea (PTU, specific inhibitor of PO), respectively. Phosphate buffer (50 mM sodium phosphate, pH 6.5) was supplied to adjust the final volume of the reaction mixtures to 110 μL. After incubation for 1, 3 and 6 h at room temperature, the total viral gDNA in each sample was extracted as described above. The numbers of viral DNA copies in each mixture were quantified with a standard curve that was generated from a series of diluted plasmids containing the fragment encoding AcMNPV *ODV–e56* (**Figure S1**) (41).

To investigate the effect of serpin–4 on melanization, nickel–nitrilotriacetic acid (Ni–NTA) agarose beads (Qiagen, Hilden, Germany) were incubated with recombinant serpin–4 overnight at 4°C (recombinant GFP was used as control). The coated Ni–NTA beads were washed three times with PBS and resuspended in PBS to approximately 100 beads/μL. Then, one microliters of resuspended Ni–NTA beads was added to a 24-well plate containing 10 μL of fresh hemolymph and 450 μL of Sf9 medium. After incubation for 2 h at room temperature, the melanization of Ni-NTA beads were observed under an inverted fluorescence microscope.

### Analysis of Inhibition of Serpin–4 on PO Activity and Amidase Activity in *O. furnacalis* Hemolymph

To measure the inhibitory potential of serpin–4 on PO activity of *O. furnacalis* hemolymph, hemolymph was collected into a 1.5 mL microcentrifuge tube from the cut abdominal prolegs of fifth instar day 1 *O. furnacalis* larvae. Aliquots (1 μL) of hemolymph was incubated for 10 min at room temperature with 9 μL of purified recombinant serpin–4 at varying concentrations. The residual PO activity in the mixtures was measured as described above. Additionally, the amidase activity of the mixtures was measured using acetyl–Ile–Glu–Ala–Arg–*p*–nitroanilide (IEAR*p*NA) as colorimetric substrate. Changes in absorbance at 405 nm were monitored in a microplate reader (Bio-Tek Instrument, Inc.). One unit of amidase activity was defined as the amount of enzyme producing an increase in absorbance (Δ*A*_405_) of 0.001 per min (39).

### Analysis of Inhibition of Serpin–4 on SP1, SP13 and SP105

To investigate whether the inhibition of PO activity by serpin-4 was due to it inhibiting SP1, SP13, and SP105 which were all involved in PPO activation (38, 39), we firstly checked whether serpin-4 could form an SDS-stable complex with one of these three serine proteases. Recombinant proSP1_Xa_, proSP13_Xa_ or proSP105_Xa_ (200 ng) were activated by Factor Xa as described previously (38, 39), and then mixed with purified recombinant serpin–4 at molar ratio of 1:1 or 1:10 (proSPs:rserpin–4). In control samples, proSPs or Factor Xa was omitted. After incubation at 37°C for 15 min, the reaction mixtures were treated with 5 × SDS sample buffer containing dithiothreitol (DTT) at 95°C for 5 min and subjected to 10% SDS–polyacrylamide gel electrophoresis (SDS–PAGE) and immunoblot analysis with mouse anti–His (1:2,000) or rabbit anti–serpin–4 (1:2,000) as the primary antibodies. Antibody binding was visualized using alkaline phosphate–conjugated horse anti–mouse (1:3,000) or goat anti–rabbit (1:3,000) and 5–bromo–4–chloro–3–indolyl phosphate/nitro blue tetrazolium (BCIP/NBT) staining buffer containing 165 mg/mL BCIP and 330 mg/mL NBT in 100 mM Tris (pH 9.5), 150 mM NaCl, and 5 mM MgCl_2_.

Furthermore, we analyzed the inhibitory potential of serpin-4 on the activities of SP1, SP13, and SP105 cleaving the respective substrate. Factor Xa-activated SP1, SP13 or SP105 (200 ng) was mixed with serpin–4 at a molar ratio of 1:1 or 1:10 (proSPs:serpin–4). After incubation at 37°C for 15 min, 200 ng of proSP13 (for SP1’s cleavage) or OfPPO2 (for SP13 and SP105) was added to the reaction mixtures and incubated at 37°C for another 15 min. The mixtures were separated with 10% or 8% SDS–PAGE and subjected to immunoblot analysis using antiserum against the mouse anti–His (1:2,000) or rabbit anti–PPO2 (1:2,000).

## Results

### AcMNPV Infection Suppressed PO Activity in *O. furnacalis* Hemolymph and Induced Serpin–4 Expression

**Figure 1:**
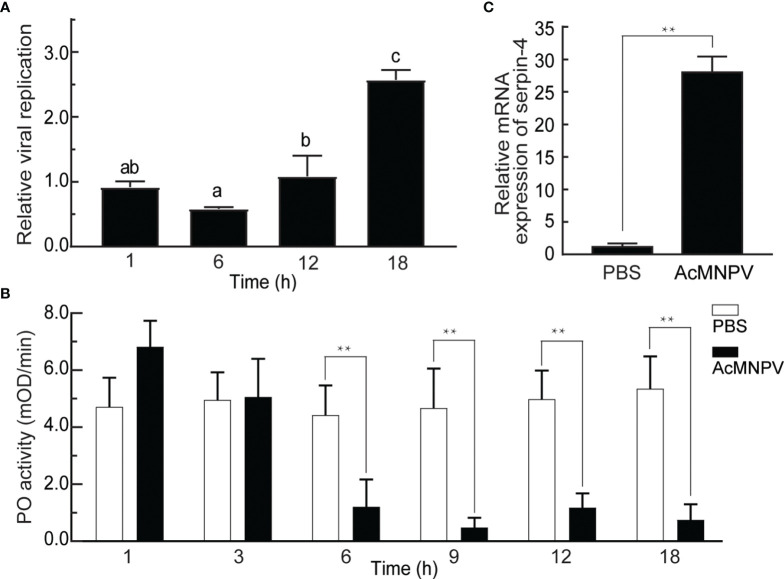
Relationship between AcMNPV infection and the innate immune response in *O. furnacalis*. **(A)** Analysis of viral replication after AcMNPV infection. Viral gDNA was extracted from larvae at 1/6/12/18 h after infection, and the relative viral amounts were determined by qRT–PCR. *O. furnacalis* ribosomal protein L8 (*rpL8*) was used as the internal control. The bars represented the mean ± S.D. (*n* = 3). Different marked letters indicated means that were significantly different (one–way ANOVA followed by Tukey’s multiple comparisons test, *P* < 0.05). **(B)** Analysis of PO activity after AcMNPV infection. Hemolymph (1 μL) collected from virus-infected or control larvae was incubated for 10 min at room temperature. PO activity was monitored using dopamine as substrate. The bars represented the mean ± S.D. (*n* = 3). Statistical significance was determined using Sidak’s multiple comparisons test (*P < 0.05, **P < 0.01). **(C)** Analysis of expression of *serpin–4* after AcMNPV infection. Fifth–instar larvae were injected with AcMNPV. The transcript level of *serpin–4* was assayed by qRT–PCR three hours later, and *rpL8* was used as an internal standard. The bars represented the mean ± S.D. (*n* = 3). Asterisks indicated means that were significantly different (unpaired t test, two–tailed, **P < 0.01).

As a first step to investigate the interaction between entomopathogenic virus AcMNPV and the host *O. furnacalis*, we infected *O. furnacalis* larvae with AcMNPV and measured the viral gDNA copies 1, 6, 12, and 18 hours post infection (hpi). As shown in **Figure 1A**, the gDNA amounts of AcMNPV remained unchanged within 12 hours after infection, but increased significantly at 18 hpi. Meanwhile, we checked PO activities of *O. furnacalis* hemolymph after AcMNPV infection. PO activity kept unchanged within 3 hours after infection, and began to decrease significantly at 6 hpi. Until 18 hpi, PO activity was still suppressed significantly (**Figure 1B**, **S2A**). It suggested that AcMNPV infection reduced PO activity in *O. furnacalis* hemolymph, and this suppression might facilitate the viral replication in the host.

On the other hand, PPO activation was regulated by the serpin(s) in insects (44). We speculated that the decrease of PO activity upon AcMNPV infection was related to the serpin(s) in *O. furnacalis*, and then checked mRNA expression of several transcripts encoding potential serpins, including CL7904.Contig2 (*serpin-3*), CL9195.Contig5, and CL5354.Contig1 (*serpin-6*) (37). As shown in **Figure 1C** and **S2B**, the abundance of 3 transcripts all increased significantly in the larvae challenged with AcMNPV. The transcript level of CL9195.Contig5 had the largest increase, up to around 25 folds 3 h after AcMNPV infection. Therefore, we only focused on CL9195.Contig5 in the studies that followed. This transcript was named as *serpin-4*.

### Molecular Cloning and Sequence Analysis of Serpin–4

The cDNA fragments encoding the entire coding region of *serpin-4* were amplified by PCR using specific primers designed based on the cDNA sequence of CL9195.Contig5 from our previous *O. furnacalis* transciptome (37). The obtained cDNA sequence of *serpin–4* (GenBank^TM^ accession number ON323051) contained a 1,242–bp open reading frame. The conceptual protein deduced from nucleotide sequence consisted of 413 amino acid residues, including a predicted 18–residue secretion signal peptide. The calculated molecular weight and the theoretical isoelectric point of the mature protein was 44.7 kDa and 6.63, respectively (**Figure S3**). Phylogenetic analysis showed that *O. furnacalis* serpin–4 clustered together with *B. mori* serpin–4, *M. sexta* serpin–4, *Operophtera brumata* serpin–4 and *Plutella xylostella* serpin–4, with the bootstrap value of 100 (**Figure S4**). Among them, *M. sexta* serpin–4 has been verified experimentally to inhibit PPO activation by inhibiting at least 4 target serine proteases (27, 45). Therefore, we predicted that *O. furnacalis* serpin–4 could also function as an inhibitor of melanization response.

### Expression Profiles of *O. furnacalis* Serpin–4

**Figure 2:**
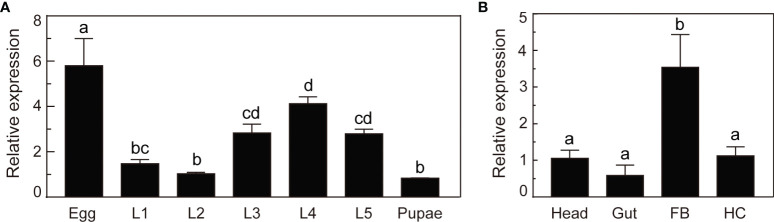
Expression profile analysis of *O. furnacalis serpin–4*. **(A)** qRT–PCR analysis of *O. furnacalis serpin–4* transcripts at different stages of development. RNA was extracted from eggs, first–instar larvae (L1), second–instar larvae (L2), third–instar larvae (L3), fourth–instar larvae (L4), fifth–instar larvae (L5) and pupae. **(B)** qRT–PCR analysis of *O. furnacalis serpin–4* transcripts in different tissues. RNA was extracted from the head, gut, fat body (FB) and hemocytes (HC). The bars represented the mean ± S.D. (*n* = 3). The *rpL8* gene was used as an internal standard to indicate a consistent total mRNA amount. Bars labeled with different letters were significantly different (one–way ANOVA, followed by Tukey’s multiple comparisons test, *P* < 0.05).

We analyzed the mRNA levels of *O. furnacalis serpin–4* in various developmental stages or different tissues using qRT–PCR methods. As shown in **Figure 2A**, *serpin–4* transcripts in eggs were significantly more than that in other developmental stages. The mRNA level of *serpin–4* remained consistent in the third, fourth and fifth instar larvae, but was significantly higher than that in the first and second instar larvae and pupae. In different tissues, *serpin–4* was expressed at significantly higher levels in fat bodies than in the head, gut and hemocytes (**Figure 2B**).

### Production and Purification of Recombinant Serpin–4 and GFP

**Figure 3:**
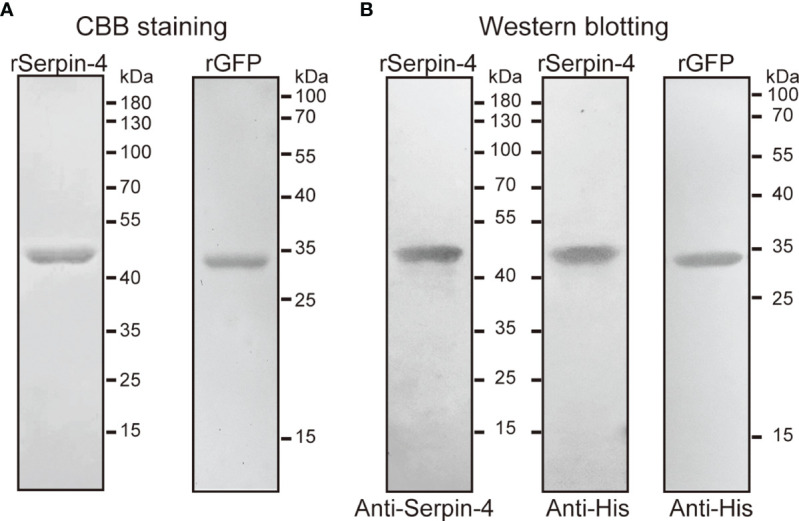
SDS–PAGE **(A)** and immunoblot analysis **(B)** of purified recombinant serpin–4 and GFP. The purified recombinant serpin–4 (250 ng) or recombinant GFP (250 ng) was treated with SDS sample buffer containing DTT, separated by 10% or 15% SDS–PAGE and subjected to Coomassie brilliant blue staining or immunoblotting with anti–His or anti–serpin–4 as primary antibodies.

In order to investigate the function of serpin–4, we produced the recombinant serpin–4 protein with an amino-terminal hexahistidine tag. SDS–PAGE analysis indicated that purified rSerpin–4 had an apparent molecular mass of 45 kDa, approximately consistent with that predicted based on its cDNA sequence (**Figure 3A**). Recombinant serpin–4 was clearly recognized by the antibodies against *O. furnacalis* serpin-4 and the commercial anti–His serum in immunoblotting analysis (**Figure 3B**). Additionally, we produced the recombinant GFP protein as a control. It had an apparent mass of approximately 34 kDa, and was recognized as a single band by anti–His serum (**Figure 3**).

### Effects of Serpin–4 on AcMNPV Infection and *O. furnacalis* Melanization

**Figure 4:**
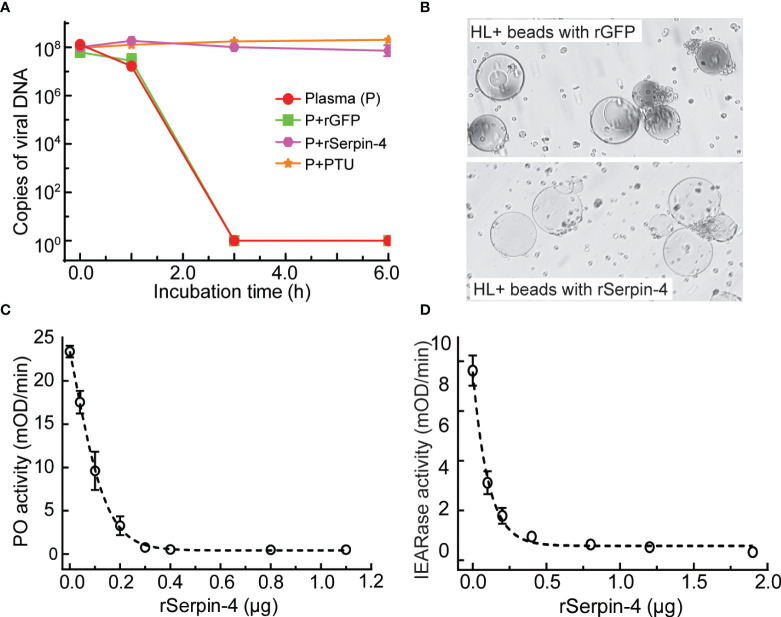
Inhibition analysis of melanization by *O. furnacalis* serpin–4. **(A)** Determination of gDNA copy numbers of AcMNPV in plasma incubated with inhibitors. AcMNPV was mixed with plasma, plasma plus PTU (P+PTU), plasma plus recombinant GFP (P+rGFP) or plasma plus recombinant serpin–4 (P+rSerpin–4). The mixtures were incubated at room temperature for 0, 1, 3, or 6 h, and the numbers of gDNA copies were determined by qRT–PCR. The data points represented the mean ± S.D. (*n* = 3). **(B)** Serpin–4 suppressed the melanization of Ni–NTA agarose beads. Ni–NTA agarose beads coated with recombinant GFP or serpin–4 were incubated with hemolymph from *O. furnacalis* larvae. The melanized beads were observed and photographed under a microscopy after 2 h of incubation. **(C)** Inhibition of PO activation by serpin–4. Hemolymph (1 μL) collected from fifth instar larvae was incubated for 10 min at room temperature with purified recombinant serpin–4 at different concentrations. PO activity was monitored using dopamine as substrate. The bars represented the mean ± S.D. (*n* = 3). **(D)** Inhibition of IEARase activation by serpin–4. Hemolymph (1 μL) collected from fifth-instar larvae was incubated with purified recombinant serpin–4 at different concentrations. The residual IEARase activity of hemolymph was plotted as the mean ± S.D. (*n* = 3).

To evaluate the effects of serpin-regulated hemolymph melanization on virus infection, we incubated AcMNPV with plasma only, or plasma plus rGFP, or plasma plus rSerpin–4, or plasma plus PTU (PTU blocks the melanization by specifically inhibiting PO) for 1, 3, and 6 h, and determined the viral gDNA copies. After 3 h incubation, the virus copies in the sample containing plasma only or plasma plus rGFP decreased to be nearly undetectable (**Figure 4A**). However, when PTU was incubated together with plasma and virus, the viral DNA copies was unchanged even after incubation for 6 h. Similar results were observed when virus was incubated with plasma together with the recombinant serpin-4 (**Figure 4A**). These results demonstrated that melanization could reduce the virus copies *in vitro* and serpin-4 worked like PTU to inhibit the melanization of hemolymph.

We further performed *in vitro* experiments to test the potential of serpin-4 inhibiting melanization. We coated Ni-NTA agarose beads with recombinant serpin-4 or GFP (as a control), and then incubated with *O. furnacalis* hemolymph. After 2 h, GFP–coated beads turned black. However, the beads coated with rSerpin-4 had no change (**Figure 4B**).

Hemolymph melanization was accompanied by induced PO activity (20). Inhibition of melanization by serpin-4 inferred that it could suppress PO activity in the hemolymph. To test this hypothesis, we measured the PO activity after the incubation of *O. furnacalis* hemolymph with different amounts of recombinant serpin–4. Serpin–4 inhibited PPO activation in a concentration–dependent manner (**Figure 4C**). It blocked PPO activation by 50% at 10 μg/mL and 95% at 30 μg/mL. On the other hand, PPO activation was mediated by multiple serine proteases, some of which exhibit IEARase activity (cleaving after arginine residue in IEAR*p*NA substrate). Thus, we examined the IEARase activity of hemolymph with the addition of different amounts of recombinant serpin–4. As the concentration of rSerpin-4 in the reaction mixtures increased, the IEARase activity gradually declined (**Figure 4D**). These results indicated that serpin–4 inhibited at least one serine protease in PPO activation cascade in *O. furnacalis*.

### Formation of SDS–Stable Complexes Between rSerpin-4 and SP1, SP13 and SP105

In previous studies, we demonstrated that two serine proteases (SP13 and SP105) acted as prophenoloxidase-activating protease in PPO activation pathway in *O. furnacalis*, and proSP13 was cleaved and activated by another serine protease (SP1) (38, 39). To reveal which protease serpin–4 inhibited in blocking PPO activation, we firstly checked whether serpin–4 could form SDS–stable, high molecular weight complex with anyone of these three proteases because the formation of such a complex was a characteristic feature for serpin to inhibit its target protease (44).

**Figure 5:**
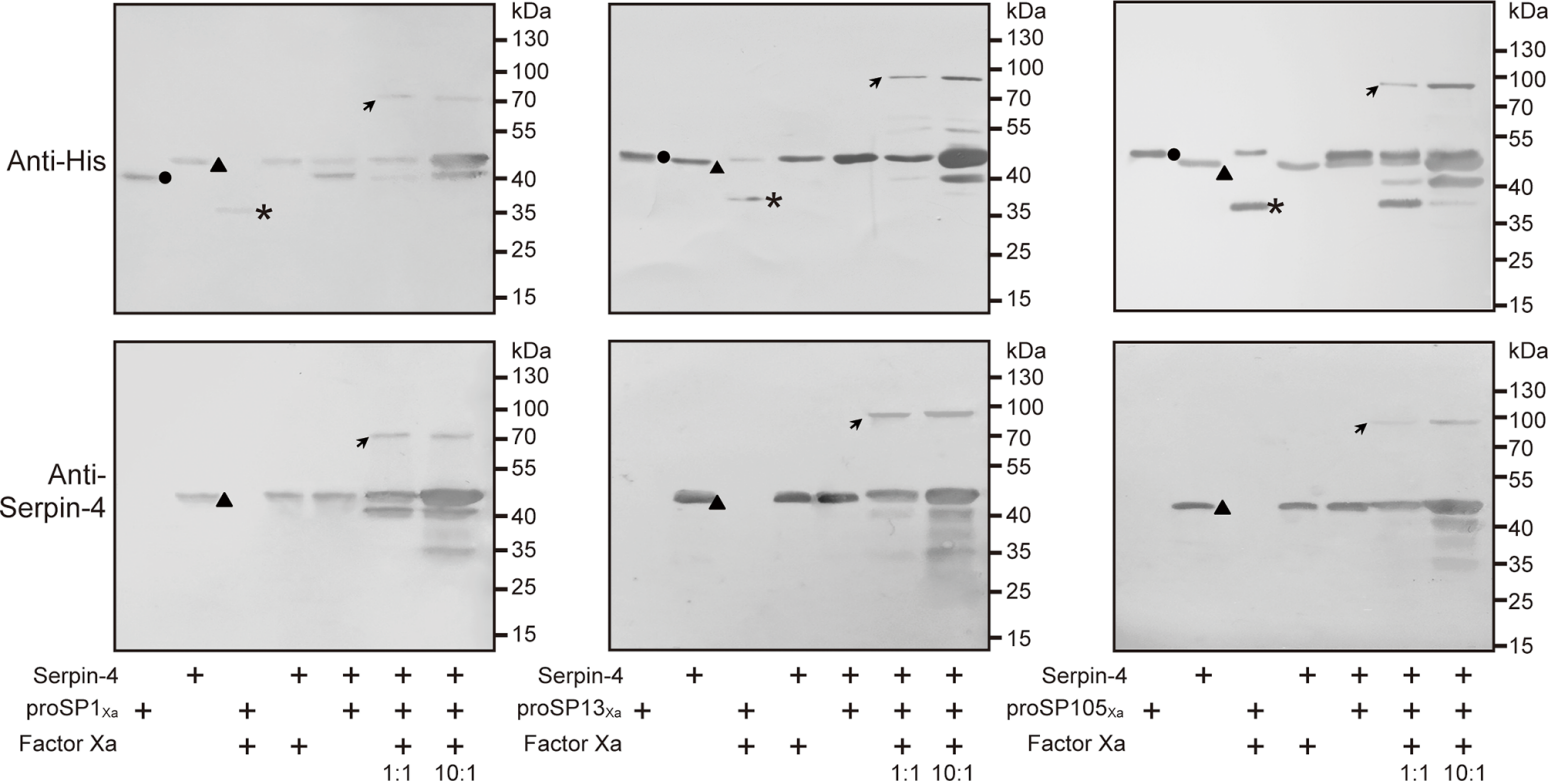
Detection of covalent complex formation between serpin-3 and three serine proteases. 200 ng of proSP1_Xa_, proSP13_Xa_ or proSP105_Xa_ was activated by Factor Xa, respectively, and incubated with purified serpin–4 at a molar ratio of 1:1 or 10:1 (serpin–4:SP_Xa_) at 37°C for 15 min. The samples were subjected to 10% SDS–PAGE and immunoblot analysis using antiserum against His (*upper panel*) or *O. furnacalis* serpin–4 (*lower panel*) as primary antibodies. The sizes and positions of the molecular mass standards were indicated to the right of each blot. Circles, proSP_Xa_; triangles, serpin–4; asterisks, catalytic domain of proSP_Xa_; arrows, serpin–4/SP complex.

The anti–His antiserum recognized purified proSP1_Xa_, proSP13_Xa_ and proSP105_Xa_ as approximately 43 kDa, 49 kDa and 50 kDa, respectively (**Figure 5**, circles in *upper* panels). After activation by Factor Xa, the bands representing the three zymogens disappeared (for proSP1_Xa_) or decreased in intensities (for proSP13_Xa_ and proSP105_Xa_). Meanwhile, a new band with the apparent molecular weight of 34 kDa, 34 kDa and 35 kDa, appeared, which corresponded to the catalytic domain of proSP1_Xa_, proSP13_Xa_ and proSP105_Xa_, respectively (**Figure 5**, asterisks in *upper* panels). When rSerpin–4 was mixed with Factor Xa alone or proSP1_Xa_/proSP13_Xa_/proSP105_Xa_ zymogen, no change was observed. However, when rSerpin-4 was mixed with Factor Xa-activated SP1_Xa_, SP13_Xa_ and SP105_Xa_, respectively, the band corresponding to the catalytic domain disappeared, and a new immunoreactive band at ~ 80 kDa (for SP1_Xa_) or ~90 kDa position (for SP13_Xa_ and SP105_Xa_) was detected, which was the expected size of a serpin-4/SP complex (**Figure 5**, arrows in *upper* panel). This band with high molecular mass was more abundant when the molar ratio of serpin-4 to SPs increased from 1:1 to 10:1 (**Figure 5**, *upper* panel). Moreover, these complexes were also recognized by antibody against serpin–4 (**Figure 5**, arrows in *lower* panel). It indicated that serpin-4 could form a covalent complex with each of SP1, SP13, and SP105 *in vitro*.

### Serpin–4 Prevented SP1_Xa_, SP13_Xa_ and SP105_Xa_ From Cleaving Its Respective Downstream Substrate

**Figure 6:**
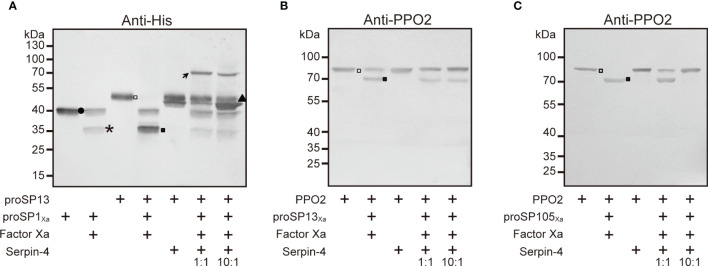
Serpin–4 prevented SP1 **(A)**, SP13 **(B)** or SP105 **(C)** from cleaving its respective downstream protease. Factor Xa–activated SP_Xa_ (200 ng) was combined with a 1- or 10-fold molar excess of serpin–4 and then incubated with recombinant proSP13 (200 ng) or OfPPO2 (200 ng) at 37°C for 15 min. The mixtures were subjected to 10% or 7.5% SDS–PAGE and immunoblotting using antiserum against His or PPO2. Circle, proSP13Xa; asterisk, catalytic domain of proSP1_Xa_, proSP13_Xa_ or proSP105_Xa_; triangle, serpin–4; hollow square, proSP13 or PPO2 zymogen; solid square, activated SP13 or PO2.

Our previous work indicated that SP1, SP13, and SP105 could cleave *O. furnacalis* proSP13, PPO2, PPO2, respectively (38, 39). If SP1/SP13/SP105 could be inhibited by serpin-4, the cleavage of their respective substrate would be theoretically suppressed in the presence of serpin-4. To test this hypothesis, we incubated Factor Xa–activated SPs with their substrates (SP1_Xa_ and proSP13; SP13_Xa_ and PPO2; SP105_Xa_ and PPO2) in the absence or presence of serpin–4. As shown in **Figure 6A**, when proSP13 was incubated with Factor Xa-activated SP1_Xa_, the ~49-kDa band corresponding to proSP13 zymogen (hollow square in **Figure 6A**) disappeared, and a ~34-kDa band corresponding to the cleaved catalytic domain of proSP13 showed up (solid square in **Figure 6A**). When Factor Xa-activated SP1_Xa_ was pre-treated with serpin–4 before mixed with proSP13, proSP13 was clearly detected and the ~34-kDa band indicating the cleavage of proSP13 became faint. Pre-treatment of more serpin-4 resulted in stronger inhibition on the cleavage of proSP13 by SP1_Xa_ (**Figure 6A**). Similarly, when Factor Xa-activated SP13_Xa_ was pre-treated with serpin–4, the processing of PPO2 by SP13_Xa_ was partly inhibited (**Figure 6B** and **Figure S5A**). Especially when Factor Xa-activated SP105_Xa_ was pre-treated with serpin–4 at a molar ration of 1:10, all added PPO2 was recognized as ~80-kDa zymogen band and no cleaved PO2 was detected (**Figure 6C** and **Figure S5B**).

## Discussion

The understanding of the immune interaction between entomopathogenic viruses and their insect hosts is incomplete. The insect, such as a serious pest *O. furnacalis*, employs its own immune response including melanization reaction to defend against the microbial infection. On the other hand, entomopathogenic virus, such as AcMNPV, suppresses *O. furnacalis* immunity and finally kills it. Comprehensive understanding of the biochemical mechanisms involved in the crosstalk between *O. furnacalis* and AcMNPV would improve the killing effects of AcMNPV, and further help to develop a new strategy on controlling *O. furnacalis*. Here, we investigated the interaction between AcMNPV infection and *O. furnacalis* melanization, and discovered that *O. furnaclais* serpin-4 facilitated AcMNPV infection by inhibiting the melanization. We further revealed serpin-4 performed the inhibitory function possibly by blocking its target proteases, SP1, SP13, and SP105.

Upon the viral infection, insects rely on several defenses including RNA interference (RNAi), Jak/STAT signaling pathway, apoptosis and autophagy to restrict viral replication and dissemination (46–48). Here we discovered that AcMNPV infection resulted in a significant decrease in PO activity of *O. furnacalis* hemolymph from 6 hpi to 18 hpi or possibly longer (**Figure 1B**). Meanwhile, viral copies decreased significantly when AcMNPV was incubated with *O. furnacalis* plasma only *in vitro*, but remained unchanged when incubated with plasma and PTU which inhibited the melanization of plasma (**Figure 4A**). It suggested hemolymph melanization in *O. furnacalis* was related to AcMNPV replication and infection. Similarly, upon AcMNPV infection, PO activity decreased and viral copies significantly increased in susceptible silkworm strains p50. Instead, PO activity increased and viral copies kept unchanged in resistant strain C108 (49). In *Aedes albopictus* - derived U4.4 cell, more cells were infected by Semliki Forest virus when PO activity of the conditioned medium was blocked (11, 50). 5, 6-dihydroxyindole (DHI), a reactive compound generated by PO, and its spontaneous oxidation products were active against viruses (51). It possibly explained why AcMNPV infection was associated with PO–catalyzed melanization in *O. furnacalis*.

On the other hand, PO–catalyzed melanization is mediated by a series of sequentially activated serine proteases and regulated by serpin superfamily (14). In this work, we identified a novel serpin transcript, *O. furnacalis* serpin-4, and illustrated that the melanization of the beads coated with recombinant serpin-4 were obviously restrained (**Figure 4B**). Recombinant serpin-4 protein inhibited PO activity and IEARase activity of *O. furnacalis* hemolymph in a concentration-dependent manner (**Figures 4C, D**). Furthermore, it was interesting that the number of AcMNPV copies significantly increased when the melanization of plasma was suppressed by serpin-4 (**Figure 4A**). It suggested AcMNPV replication was indeed associated with melanization, and as well inferred that *O. furnacalis* serpin-4 had the potential to inhibit the melanization. Similar results were found in other insects. Knockdown of *serpin-5* or *serpin-9* in *H. armigera* with RNAi significantly increased PO activity of hemolymph and dramatically reduced the number of HearNPV DNA copies (26). In *B. mori*, the copy numbers of viral genomic DNA also decreased in *Bmserpin2*-depleted hemolymph (34). Therefore, we concluded that the suppression of melanization caused by serpin responded to viral infection, and depletion of *serpin* might enhance the virulence of entomopathogenic virus. In our study, *O. furnacalis serpin-3* and *serpin-6* was also induced upon AcMNPV infection besides *serpin-4* (**Figure S2B**). The function of *serpin-6* was completely unknown so far. Serpin-3 has been clarified to regulate the melanization of *O. furnacalis* hemolymph (32). Future work would test whether serpin-3 could also facilitate virus infection by inhibiting melanization response. We further deciphered the mechanism of serpin-4 regulating *O. furnacalis* melanization reaction. Recombinant serpin–4 formed covalent complexes with three serine proteases (SP1, SP13 and SP105) which were all involved in melanization pathway (**Figure 5**) (38, 39). It was consistent with the characteristic feature of serpin in which it forms covalent complexes with its target protease(s) (44). Such serpin/protease regulatory unites were reported as serpin-12/HP14 (30), serpin-5/HP6 (28) and serpin-5/HP1 (27) in *M. sexta*, serpin-5/cSP4, serpin-9/cSP6, and serpin-9/cSP29 in *H. armigera* (26). Complex formation made serpin covalently linked to the target protease, which was therefore irreversibly inhibited (19). Here, we also observed serpin-4 strongly prevented SP1, SP13 and SP105 from cleaving their cognate downstream protease - proSP13, PPO2, and PPO2, respectively (**Figure 6**). Therefore, we speculated serpin-4 regulated the melanization of *O. furnacalis* hemolymph in this way, and further made for the virus infection. It is reasonable that insects are infected more easily by pathogens or viruses when its immune system is weakened by the negative inhibitors such as serpins. For example, expression of a serpin homologue (Hesp018) in AcMNPV increased the viral virulence and resulted in an increased mortality of infected *Trichoplusia ni* (35). In *Galleria mellonella* and *Myzus persicae*, serpin-expressing *B. bassiana* strain suppressed PO activation in host hemolymph and exhibited higher virulence (52).

For entomopathogenic microbes, they evolved various strategies to overcome host immunity, for example, inhibit the melanization reaction. Some produced antibiotics to suppress melanization. Entomopathogenic bacterium *Photorhabdus luminescens* released a small-molecule antibiotic to directly block PO activity (53). Some expressed its own viral protein to inhibit the host PAPs, for example, Egf1.0 and Egf1.5 produced in *Microplitis demolitor* bracovirus suppressed the processing of host PPO by PAP and SPH (54). Some expressed serpin-like protein to target at host serine protease, for example, parasitoid wasp *Pteromalus puparum* secreted serpin isoform *Pp*S1V to inhibit host PPO activation by forming complexes with host PrHP8 and PrPAP1 (55). In our study, entomopathogenic virus AcMNPV employed host serpin-4 to suppress host melanization by inhibiting its potential target proteases SP1, SP13 and SP105. However, it is unknown how AcMNPN induced the expression of *O. furnacalis* serpin–4 and whether other serpin(s) also contributed to AcMNPV infection. More investigation is ongoing. The findings would provide a theoretical basis for better controlling agricultural pests with entomopathogenic virus.

## Data Availability Statement

The original contributions presented in the study are included in the article/**Supplementary Material**. Further inquiries can be directed to the corresponding author.

## Author Contributions

CA and JJ contributed to the conception and design of the experiments. JJ, SZ, and DS performed the experiments. JJ and LW processed the data. JJ and CA wrote the article. All authors contributed to the article and approved the submitted version.

## Funding

This work was supported by National Key R&D Program of China (2019YFE0120400), grants 31672361 from the National Natural Science Foundation of China, and the Youth Program of Natural Science Foundation of Jiangsu Province (BK20190959).

## Conflict of Interest

The authors declare that the research was conducted in the absence of any commercial or financial relationships that could be construed as a potential conflict of interest.

## Publisher’s Note

All claims expressed in this article are solely those of the authors and do not necessarily represent those of their affiliated organizations, or those of the publisher, the editors and the reviewers. Any product that may be evaluated in this article, or claim that may be made by its manufacturer, is not guaranteed or endorsed by the publisher.

## Supplementary Material

Supplementary Figure 1 Standard curve for the quantification of viral copy numbers by qRT-PCR. The CT values were plotted as X-axis. The logarithm of viral copy numbers was indicated on Y-axis. The standard curve parameters calculation was shown above the curve.

Supplementary Figure 2 Analysis of PO activity **(A)** and mRNA expression **(B)** after AcMNPV infection. **(A)** Hemolymph (1 μL) collected from *O. furnacalis* larvae infected by AcMNPV at different concentrations was incubated for 10 min at room temperature. PO activity was monitored using dopamine as a substrate. The bars represented mean ± S.D. (*n* = 3). Statistical significance was determined using Tukey’s multiple comparisons test (**P* < 0.05, ***P* < 0.01). **(B)** Fifth–instar larvae were injected with AcMNPV. The transcript levels of *O. furnacalis serpin–3*, *serpin–4* and *serpin–6* were assayed by qRT–PCR. *rpL8* was used as an internal standard to normalize the templates. The bars represented the mean ± S.D. (*n* = 3). Asterisks indicated means that were significantly different (unpaired t test, two–tailed, **P* < 0.05, ***P* < 0.01).

Supplementary Figure 3 Sequence analysis of serpin–4. The deduced amino acid sequence was shown below the nucleotide sequence of *O. furnacalis* serpin–4. The one–letter code for each amino acid was aligned with the second nucleotide of the corresponding codon. The stop codon was marked with an asterisk (*). The predicted secretion signal peptide was underlined and assigned negative numbers. Putative *N*–linked and *O*–linked glycosylation sites were heavily and lightly shaded, respectively. The potential RCL region was in the square box with the predicted P1 and P1’ residues in boldface. The predicted scissile peptide bond was indicated with “ǁ”.

Supplementary Figure 4 Phylogenetic analysis of *O. furnacalis* serpin–4 and serpins from other insect species. The used amino acid sequences were from *Ostrinia furnacalis* (Of, red), *Anopheles gambiae* (Ag), *Bombyx mori* (Bm), *Drosophila melanogaster* (Dm), *Manduca sexta* (Ms), *Operophtera brumata* (Ob), *Plutella xylostella* (Px), *Tenebrio molitor* (Tm). GenBank accession numbers of this specific genes were given in parentheses. The clade that groups *O. furnacalis* serpin-4 with other serpin-4s was shaded in blue. The numbers at the nodes indicated the bootstrap values as percentages of 1000 repetitions.

Supplementary Figure 5 Serpin–4 inhibited the cleavage of recombinant PPO2 by SP13 **(A)** and SP105 **(B)**. Factor Xa–activated SP13_Xa_ or SP105_Xa_ (200 ng) was combined with a 1- or 10-fold molar excess of serpin–4 at 37°C for 15 min and then incubated with recombinant PPO2 (200 ng) at 37°C for another 15 min. The mixtures were subjected to 7.5% SDS–PAGE and immunoblotting using antiserum against His. Circle, proSP13_Xa_ or proSP105_Xa_; asterisk, catalytic domain of proSP13_Xa_ or proSP105_Xa_; triangle, serpin–4; hollow square, PPO2 zymogen; solid square, activated PO2.

Supplementary Table 1 Oligonucleotides primers used in this study.
