# Supplementary material for: Serpin–4 Facilitates Baculovirus Infection by Inhibiting Melanization in Asian Corn Borer, Ostrinia furnacalis (Guenée)
Source: Front Immunol. 2022 Jun 9;13:905357. doi: 10.3389/fimmu.2022.905357 (PMC9218052; doi:10.3389/fimmu.2022.905357)
Supplement: Supplementary Figure 1 — Standard curve for the quantification of viral copy numbers by qRT-PCR. The CT values were plotted as X-axis. The logarithm of viral copy numbers was indicated on Y-axis. The standard curve parameters calculation was shown above the curve. [file DataSheet_1.docx]

Supplementary Material

# Supplementary Tables

**Supplementary Table 1.** Oligonucleotides primers used in this study.

|  | Forward primers (5’-3’) | Reverse primers (5’-3’) |
| --- | --- | --- |
| qRT-PCR: | | |
| Serpin-3 | ATTGCAGCACAAATCGCCCC | GTGGGCAACTGCTGCAAACT |
| Serpin-4 | TGACTCTGAGCAAGCCAGGTT | ACAGTTCCCACCTCCGTCAC |
| Serpin-6 | GCTGGTGCTAGCCAATGCTG | CGGGTCTCCGACACGAAGAA |
| ODV-e56 | GATCTTCCTGCGGGCCAAACACT | AACAAGACCGCGCCTATCAACAAA |
| rpL8 | AAGCGAGGAACATCAGCC | GGTCTTGCCACCACGAAT |
| Amplification of mature Serpin-4, excluding signal peptide, with restriction sites for cloning in pET-28a vector: | | |
|  | CATCATCACCAGTCTGTGGGTAATTCC | GCGGCCGCTCAGTACAAAGATGGTT |
|  | ATCCATGGGCCATCATCATCATCATCAC |  |

# Supplementary Figures


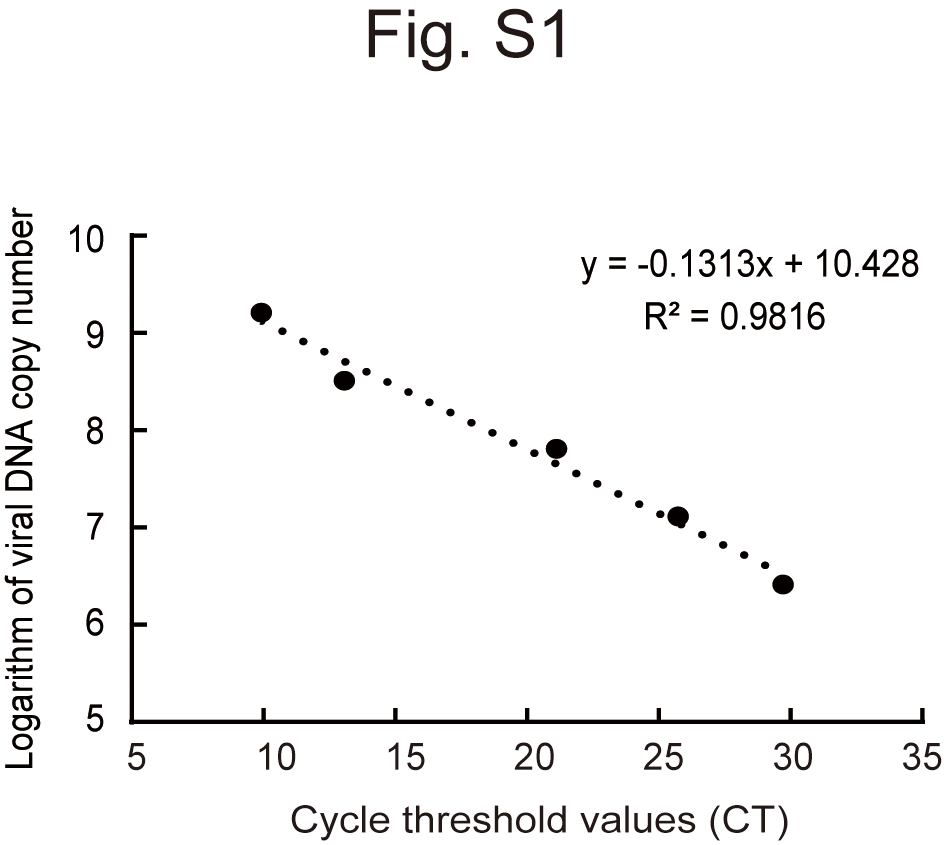


**Supplementary Figure 1.** Standard curve for the quantification of viral copy numbers by qRT-PCR. The CT values were plotted as X-axis. The logarithm of viral copy numbers was indicated on Y-axis. The standard curve parameters calculation was shown above the curve.


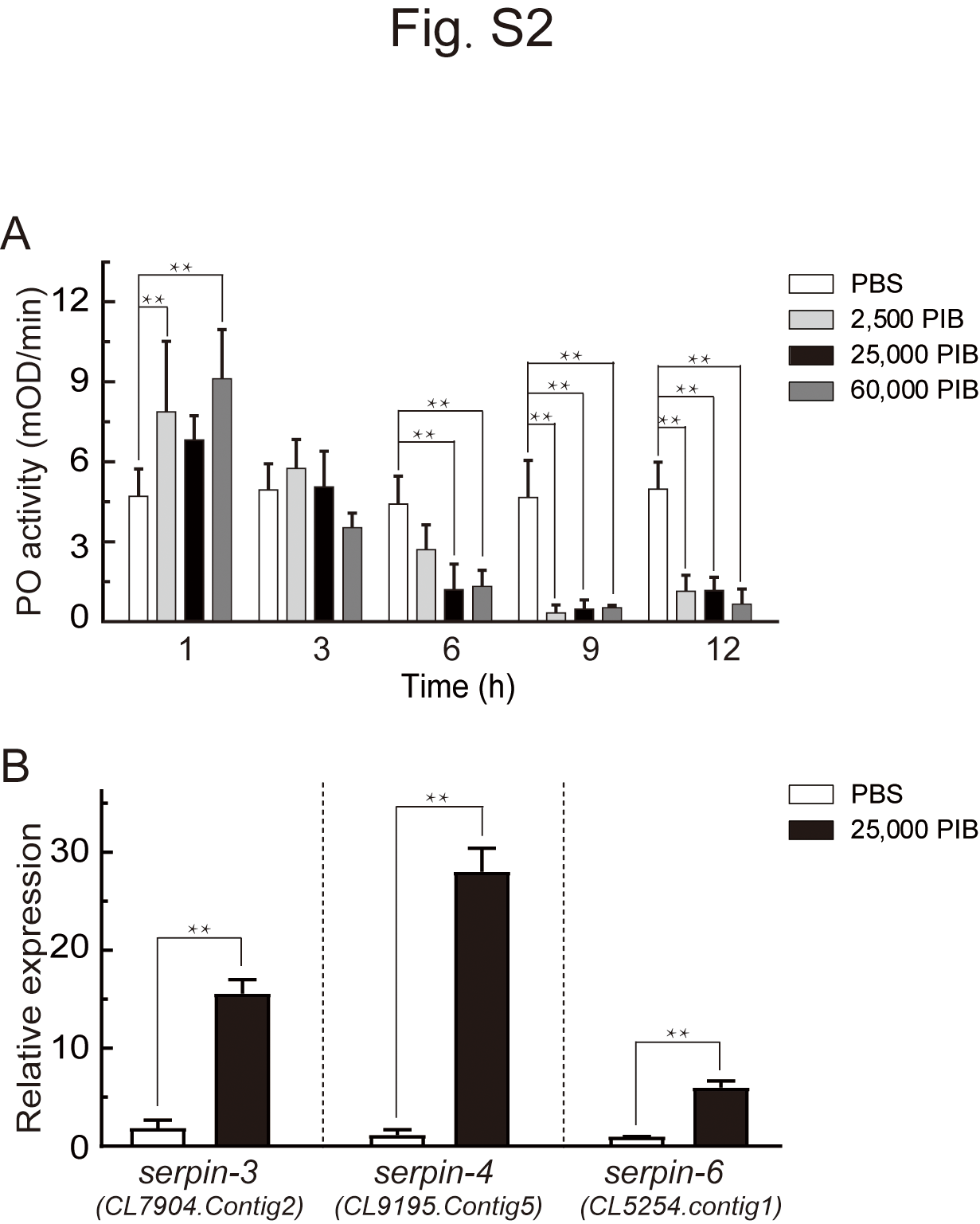


**Supplementary Figure 2.** Analysis of PO activity (A) and mRNA expression (B) after AcMNPV infection. (**A**)Hemolymph (1 μL) collected from *O. furnacalis* larvae infected by AcMNPV at different concentrations was incubated for 10 min at room temperature. PO activity was monitored using dopamine as a substrate. The bars represented mean ± S.D. (*n* = 3). Statistical significance was determined using Tukey's multiple comparisons test (* *P* < 0.05, ** *P* < 0.01). (**B**) Fifth–instar larvae were injected with AcMNPV. The transcript levels of *O. furnacalis serpin–3,* *serpin–4* and *serpin–6* were assayed by qRT–PCR. *rpL8* was used as an internal standard to normalize the templates. The bars represented the mean ± S.D. (*n* = 3). Asterisks indicated means that were significantly different (unpaired t test, two–tailed, * *P* < 0.05, ** *P* < 0.01).


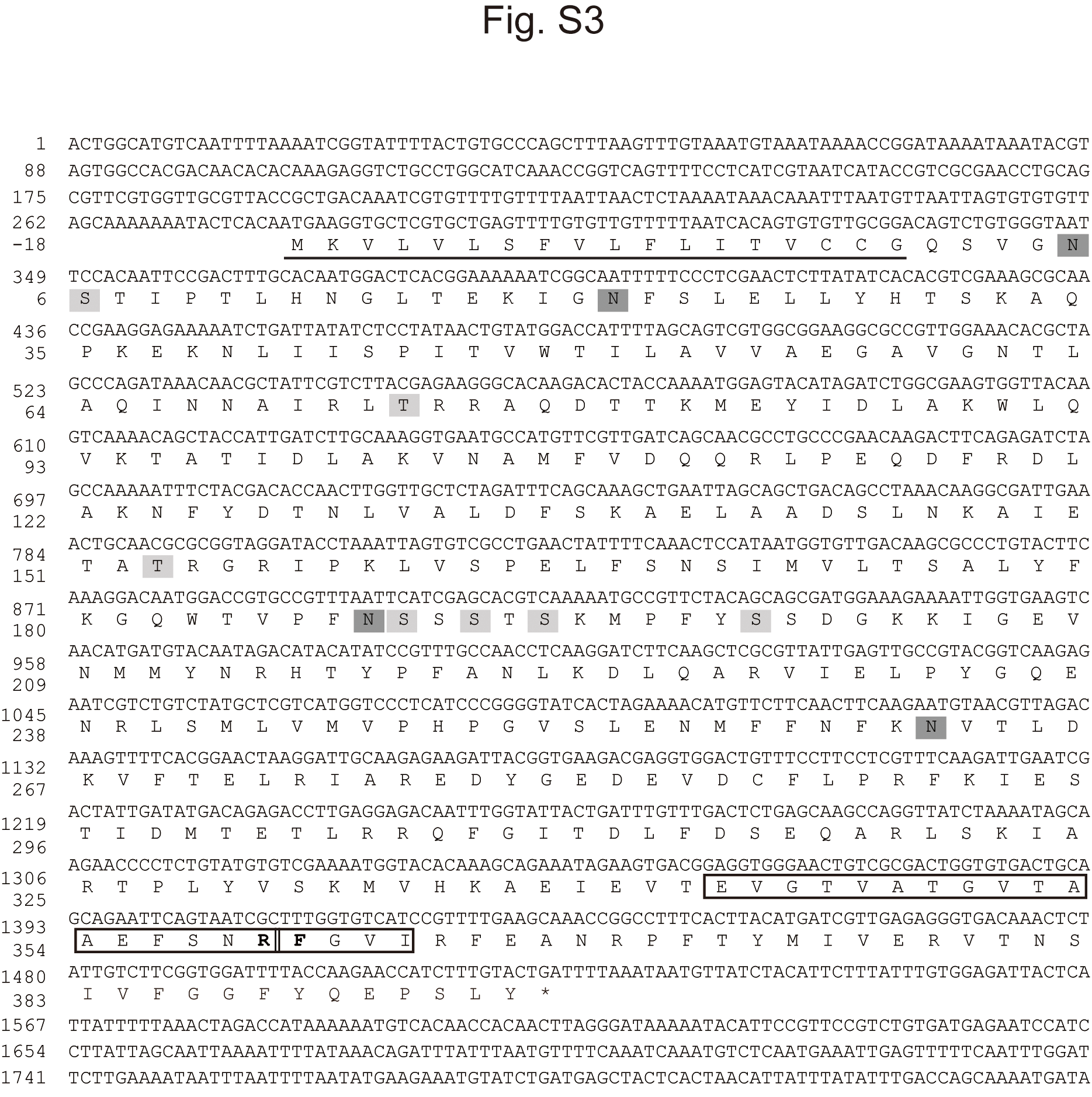


**Supplementary Figure 3.** Sequence analysis of serpin–4. The deduced amino acid sequence was shown below the nucleotide sequence of *O. furnacalis* serpin–4. The one–letter code for each amino acid was aligned with the second nucleotide of the corresponding codon. The stop codon was marked with an asterisk (*). The predicted secretion signal peptide was underlined and assigned negative numbers. Putative *N*–linked and *O*–linked glycosylation sites were heavily and lightly shaded, respectively. The potential RCL region was in the square box with the predicted P1 and P1’ residues in boldface. The predicted scissile peptide bond was indicated with “ǁ”.


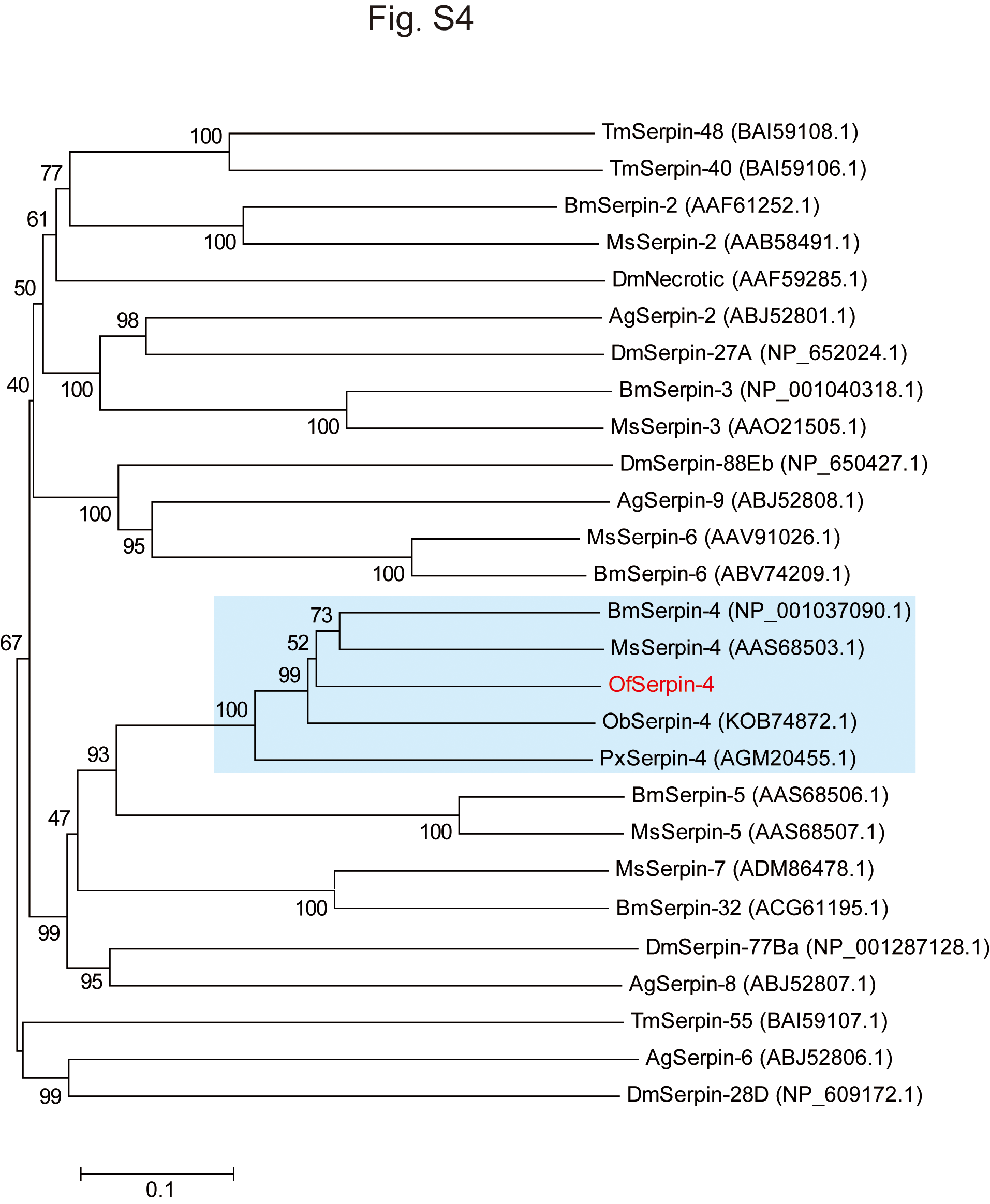


**Supplementary Figure 4.** Phylogenetic analysis of *O. furnacalis* serpin–4 and serpins from other insect species. The used amino acid sequences were from *Ostrinia* *furnacalis* (Of, red), *Anopheles gambiae* (Ag), *Bombyx mori* (Bm), *Drosophila melanogaster* (Dm), *Manduca sexta* (Ms), *Operophtera brumata* (Ob), *Plutella xylostella* (Px), *Tenebrio molitor* (Tm). GenBank accession numbers of this specific genes were given in parentheses. The clade that groups *O. furnacalis* serpin-4 with other serpin-4s was shaded in blue. The numbers at the nodes indicated the bootstrap values as percentages of 1000 repetitions.


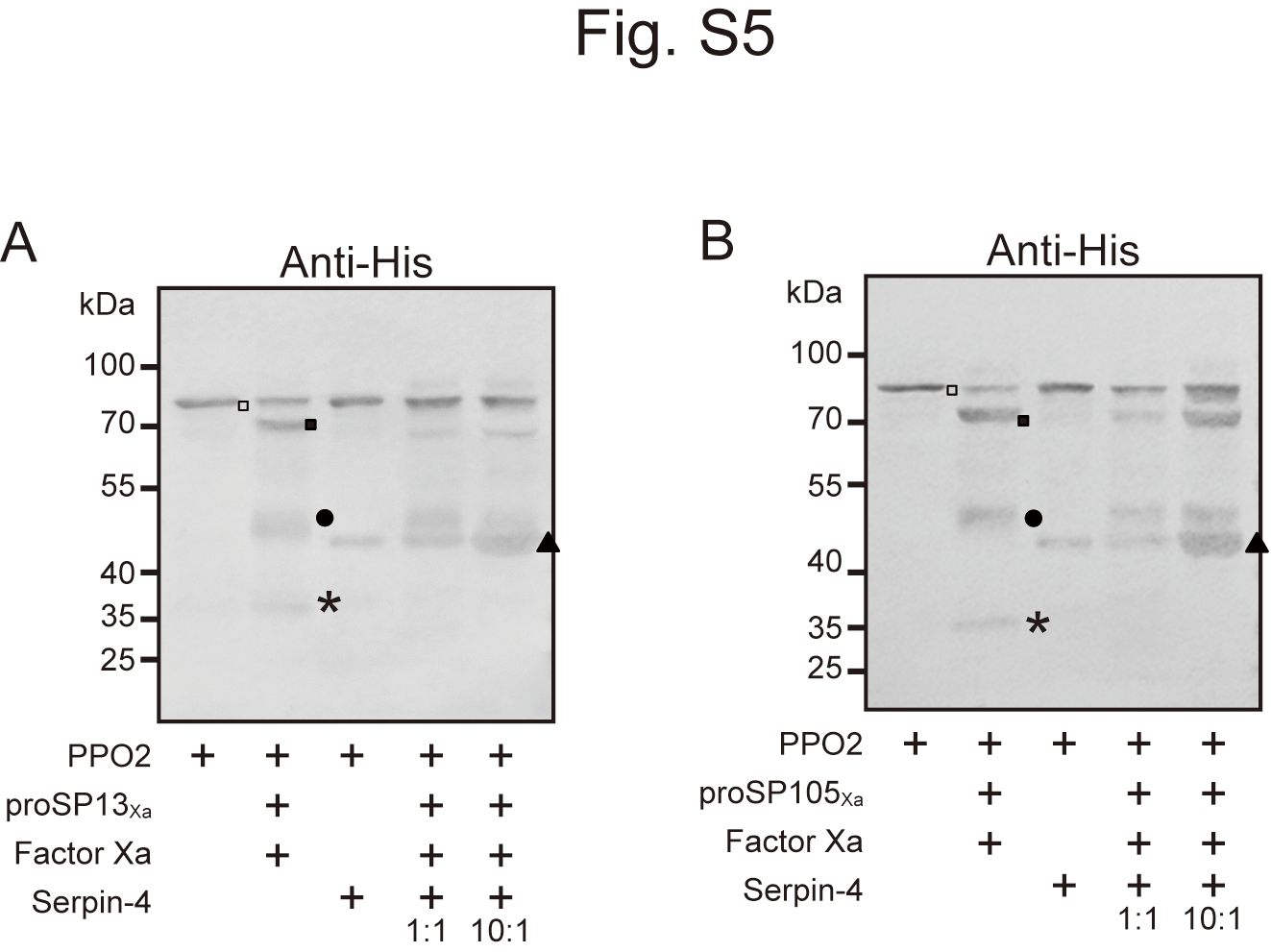


**Supplementary Figure 5.** Serpin–4 inhibited the cleavage of recombinant PPO2 by SP13 (**A**) and SP105 (**B**). Factor Xa–activated SP13_Xa_ or SP105_Xa_ (200 ng) was combined with a 1- or 10-fold molar excess of serpin–4 at 37°C for 15 min and then incubated with recombinant PPO2 (200 ng) at 37°C for another 15 min. The mixtures were subjected to 7.5% SDS–PAGE and immunoblotting using antiserum against His. Circle, proSP13_Xa_ or proSP105_Xa_; asterisk, catalytic domain of proSP13_Xa_ or proSP105_Xa_; triangle, serpin–4; hollow square, PPO2 zymogen; solid square, activated PO2.
